# Severity Status of COVID-19 and Its Associated Factors at the Nyarugenge Treatment Center in Rwanda

**DOI:** 10.7759/cureus.35627

**Published:** 2023-02-28

**Authors:** James Ntambara, Cyprien Munyanshongore, Vedaste Ndahindwa

**Affiliations:** 1 Accident and Emergency, King Faisal Hospital, Kigali, RWA; 2 School of Public Health, University of Rwanda, College of Medicine and Health Sciences, Kigali, RWA

**Keywords:** covid-19, severity status, risk factors, hospitalized patients, rwanda

## Abstract

Background

The COVID-19 pandemic has continued to be a public health emergency currently; on March 11, 2020, the World Health Organization (WHO) declared it a global pandemic. Despite the Rwanda National Health Measures that have been put in place to protect the public including lockdowns, curfew, face mask mandate, handwashing sensitization, etc., severe morbidity and mortality cases of COVID-19 are continued to be seen. Some studies have linked COVID-19 complications to its direct chain of mechanism; however, other studies have linked comorbidity or underlying disease conditions to its poor prognosis. Studies have not yet been conducted in Rwanda on the severe status of COVID-19 and its associated factors among patients. Therefore, this study aimed to assess the severe status of COVID-19 and its associated factors at the Nyarugenge Treatment Center.

Methods

A descriptive cross-sectional study was done. All patients admitted to the Nyarugenge Treatment Center from January 8, 2021, when the hospital opened, until the end of May 2021 were recruited in the study. The eligible participants were all patients who were admitted and tested positive for COVID-19 by RT-PCR method according to the Rwanda Ministry of Health criteria.

Results

All data were analyzed using the Statistical Package for the Social Sciences (SPSS) software, version 25 (IBM Corp., Armonk, NY). The number of patients admitted during the study period was 648, with a median age of 53; 45.2% of them were females, and 54.2% were males. Of these, 81.2% (526) were discharged from the hospital, while 18.8% (122) died. The proportion of severe status of COVID-19 was 42.1%. The factors that showed a risk of severe COVID-19 status were age and the number of comorbidities. Patients aged above 60 years (OR = 11.7, 95% CI: 5.35-25.67, p-value < 0.001) and those between the age of 51 and 60 (OR = 6.86, 95% CI: 2.96-15.93, p-value < 0.001) were 12 and seven times more likely to have severe COVID-19 status compared to those aged below 30 years. Having two comorbidities had twice the risk of developing a severe COVID-19 status compared to those with no comorbidity (OR = 2.13, 95% CI: 1.20-3.77, p-value < 0.001).

Conclusion

Elderly people and those with comorbidities are encouraged to obtain all standard operating procedures and comply with the vaccination program.

## Introduction

Coronavirus disease 2019 (COVID-19) is a novel coronavirus that first started to appear in Wuhan city, China, in December 2019 [1]. The virus became an epidemic in China, and the disease continued spreading profusely in all parts of Asia due to community transmission and aviation transportation, and now almost all of the globe is affected. On January 30, 2020, the World Health Organization (WHO) called nations for a public health international emergency as the virus was continuing to spread, and on March 11, 2020, the WHO declared it a global pandemic [2,3].

Over 37 million cases and one million deaths have been recorded worldwide, with 48% of all cases and 55% of all deaths being reported in the areas of America with the USA, Brazil, and Argentina being predominant. Africa has recorded (3%) 1.2 million cases and 27,000 deaths as of October 11, 2020 [2]. The disease attacks the respiratory system and causes flu-like symptoms including fever, cough, sneezing, severe pneumonia, multiple organ complications, respiratory failure, and death. SARS-CoV-2 is transmitted from human to human by direct contact with an infected person's respiratory aerosols or droplets when coughing, sneezing, or conversing in a distance less than 2 meters or by indirect contact with an infected person's immediate surroundings [1,4]. Although some articles have related factors including young population and climatic conditions with a slow spread of COVID-19 in Africa [5], cases have been increasing exponentially across the continent due to community transmission [2,6].

In Rwanda, the first case of COVID-19 was recorded on March 14, 2020, and three days later, the government incited a national lockdown, also ensuring a 14-day mandatory quarantine at all points of entry (PoE) and educating the community on public health measures, including the mandatory wearing of face masks, handwashing with soap, social distancing, and prohibited public gatherings [7]. Also, screening, testing, and contact tracing were escalated to combat the spread of the disease [4]. Since then, the cases have grown slowly with approximately a reproductive number of 0.4, and most of the cases were seen in border truck drivers at an early stage [4]. A total of 8,383 COVID-19 cases have been reported across the country with 94 deaths as of December 31, 2020 [7].

Some articles based on autopsy have reported that these people die directly from the COVID-19 chain of mechanisms and its complications rather than other factors [8]. However, other studies in China and Italy have linked old age and the presence of one or more comorbidity with a poor prognosis of COVID-19 [9,10]. Rwanda has been good in dealing with COVID-19, where the test positivity rate done through contact tracing has been at 1.4%-2% and the case fatality rate (CFR) has been at 1.1% as of January 2021 [7]. This low number of deaths in Rwanda has been attributed due to the early government interventions against COVID-19. Strong political intent, community involvement, use of technology, and public education have all been key in reducing disease spread [7,11].

Furthermore, this increase in COVID-19 cases in all age groups and among people with underlying disease conditions causes hospital settings to be overwhelmed due to patient overload, and services regarding non-communicable diseases (NCDs) may be interfered due to COVID-19 mitigation response [12]. In Rwanda, strategies to help people with NCDs to access essential care during this pandemic were available, where allowances/ passes to seek medical services for all people were granted during the national lockdown and curfew periods. However, severe cases of COVID-19 are continued to be seen. No study has been done in Rwanda yet; therefore, this study aimed to assess the severity status of COVID-19 and its associated factors at the Nyarugenge Treatment Center.

The specific objectives of this study are to (1) determine the proportion of patients with severe COVID-19 status at the Nyarugenge Treatment Center and (2) identify the risk factors associated with severe COVID-19 status at the Nyarugenge Treatment Center.

## Materials and methods

Study area description

The study was done at the Nyarugenge Treatment Center (TC). It is one of the six treatment centers for COVID-19 in Rwanda with more beds, and it also serves as the COVID-19 referral center in Rwanda. It started its activities as a COVID-19 referral center on January 8, 2021. This site is a re-location started after shifting from Kanyinya Treatment Center where COVID-19 patients were first admitted at the beginning of the pandemic in Rwanda. The facility has a capacity of 300 beds, planted with plenty of oxygen therapy, and an intensive care unit (ICU) of 140 beds. The center specializes in managing critical cases with well-equipped staff and equipment. It is located in Kigali city, Nyarugenge District, Nyamirambo Sector. Nyarugenge TC is a big center that receives almost all cases around the country from district hospitals and cases from home-based care. It also has rooms for asymptomatic, mild/moderate, severe, and critical cases. It is comprised of a multidisciplinary team mainly including physicians, specialized nurses, nutritionists, and psychotherapists.

Study design

A descriptive cross-sectional study was done on all patients admitted to the Nyarugenge Treatment Center from when the hospital opened on January 8, 2021, until the end of May 2021.

Achievement of specific objectives

The proportion of severe status of COVID-19 was calculated by taking all cases that presented with severe and critical states on admission. Indeed, the admission criteria at the hospital included the following: having clinical symptoms like fever, upper or lower respiratory symptoms, gastrointestinal (GI) symptoms, and a positive reverse time polymerase chain reaction (RT-PCR) test on throat swabs although the antigen/rapid test may be positive or negative. Discharge criteria at the Nyarugenge TC are done when there is a two-time RT-PCR-negative test, absence of fever in the last three days, and remarkable improvement of respiratory symptoms. Transfer criteria to another COVID-19 unit (post-COVID-19 follow-up) were based on a two-time PCR-negative test. All patients who were transferred from the treatment center were considered to have been discharged from the hospital. Data on the case severity were also collected.

The severity of COVID-19 cases was grouped based on the stages of COVID-19 as recommended in the Rwanda Biomedical Center (RBC)/National COVID-19 Clinical Management Guidelines (third edition, 2020) [4], where asymptomatic includes the patients with no clinical symptoms of the disease despite the confirmed diagnosis; mild or moderate when the patients have a fever, have one respiratory symptom, and are with/without signs of pneumonia on chest x-ray without the need of oxygen therapy; severe state includes the patients who have tachypnea with ≥30 c/min of respiratory rate, respiratory distress, ≤93% of oxygen saturation in the blood (SpO2) on room air, or ≤90% SpO2 on room air for children, being supplemented oxygen by facial mask or nasal prong; and critical state includes patients with any of the following symptoms: altered mental status, respiratory failure requiring mechanical ventilation, shock, and multiple organ failure that requires ICU advanced care. Data on the patient's number of comorbidities were also collected. Bivariate and multivariate analyses were performed to identify the risk factors with a severe status of COVID-19.

Study population

According to the criteria established by the Rwanda Ministry of Health (MOH) Clinical Management Guidelines for COVID-19, all patients diagnosed with COVID-19 at the Nyarugenge Treatment Center were considered [4].

Inclusion criteria and exclusion criteria

According to the RBC criteria, all COVID-19-positive patients who were admitted were included in the study. Patients who came from home-based care for outpatient care services and those who came for COVID-19 screening services were excluded from the study.

Data collection procedures

An approval letter was received from the MOH/National Health Research Committee with approval number: NHRC/2021/PROT/028. Data collection was started using hospital records from January 8, 2021, when the hospital was opened, until the end of May 2021. The data collection was done retrospectively in two weeks. The questionnaire was pretested before the start of data collection in collaboration with a medical team at the hospital. The questionnaire was adapted from the studies of Corse et al. and Osibogun et al. [13,14], which collects data on different variables. It included information on the patient's age, gender, and origin (either a referral from a district hospital or from home-based care), comorbidities, clinical signs and symptoms on admission, date of the COVID-19-positive test, vaccination status, oxygen supplement on admission, the severity of the disease, admission to ICU, and the clinical outcome including discharge or death. The severity of COVID-19 was compared to the number of comorbidities to determine the added risk of severity. Hospital admission registers for accident and emergency, ICU patient register books, cause of death certificate booklet, and discharge books were used as a source of data, and all data were kept confidential.

These register books include data on patient's demographics (i.e., names, age, gender), date of admission, date of discharge, symptoms on admission, whether on oxygen (in liters/minute), COVID-19 severity status, presence of comorbidity, and medications given on admission. The ICU register books include information about patients admitted to ICU, disposition/transfer to other wards, recovery, or death.

Data analysis

All data were entered into the Statistical Package for the Social Sciences (SPSS) software, version 25 (IBM Corp., Armonk, NY). Univariate analysis was used to calculate percentages and frequencies as well as the proportion of hospitalized cases that had a severe status of COVID-19. Bivariate analysis was used to identify the significant variables with a severe status of COVID-19, and multivariate analysis was performed considering the significant variables from bivariate analysis. Significant variables were defined as having a p-value of less than 0.05.

## Results

Table 1 shows the sociodemographic characteristics of the study participants; the median age of all patients (N = 648) admitted during the study period with a positive COVID-19 RT-PCR test was 53 years (IQR 37-69). The majority of patients (38.9%) were above the age of 60; 18.6% were between the ages of 30 and 40, 15.4% were between the ages of 41 and 50, 15.3% were between the ages of 51 and 60, and 11.8% were under the age of 30. Out of 648 patients, 54.2% were males and 45.8% were females. Most patients were Rwandans (94.6%) as shown in Table 1.

**Table 1:** Sociodemographic characteristics of the study participants N: Number of study participants.

Characteristics	Frequency (N)	Percentage (%)
Age (Median [Q1-Q3])	53.0 (37.0-69.0)	
*Age group (years)*		
<30	75	11.8
30-40	118	18.6
41-50	98	15.4
51-60	97	15.3
>60	247	38.9
*Gender*		
Female	297	45.8
Male	351	54.2
*Country of origin*		
Rwanda	613	94.6
Kenya	11	1.7
India	8	1.2
Burundi	3	0.5
Uganda	2	0.3
Democratic Republic of Congo	2	0.3
China	2	0.3
Egypt	2	0.3
France	2	0.3
Sri Lanka	1	0.2
Tanzania	1	0.2
Philippines	1	0.2

Table 2 shows that of all patients (N = 648), 58.5% presented without oxygen requirement, 40.3% required supplemental oxygen, and 0.9% came intubated on admission. Of these, 40.7% (264) had comorbidity, and 59.3% had no comorbidity. Only 2.6% were vaccinated against COVID-19, and 97.4% were not vaccinated. The commonly seen comorbidities were hypertension (21.8%), diabetes (15.3%), asthma (3.7%), and congestive heart disease (0.8%). During the study period, 1.4% of the patients were admitted to the hospital with a critical state of COVID-19, 40.7% were in a severe state, 26.5% were in a moderate state, 30.9% were in a mild state, and 0.3% were in an asymptomatic state. Of all patients, 19.9% needed ICU admission. Among the admitted patients, 81.2% were discharged, and 18.8% died.

**Table 2:** Clinical characteristics of the study participants

Clinical characteristics	Frequency	Percentage (%)
*Oxygenotherapy*		
Room air	379	58.5
Oxygenotherapy (2-15 L/min)	261	40.3
High oxygen flow	2	0.3
Intubated	6	0.9
*Comorbidity*		
Yes	264	40.7
No	384	59.3
*Types of comorbidities*		
Hypertension	141	21.8
Diabetes	101	15.6
Asthma	24	3.7
Pregnancy	17	2.6
HIV	14	2.2
Hepatitis infection	13	2.0
Chronic kidney disease	12	1.9
Cancer	11	1.7
Congestive heart disease	5	0.8
*Severity of the disease at admission*		
Asymptomatic	3	0.3
Mild	200	30.9
Moderate	172	26.5
Severe	264	40.7
Critical	9	1.4
*Vaccination status*		
Yes (1 or 2 doses)	17	2.6
No	631	97.4
*ICU admission*		
Yes	130	19.9
No	518	80.1
*Admission outcome*		
Died	122	18.8
Discharged	526	81.2

Bivariate analysis was done to assess the predictors of COVID-19 severe status. The following variables were significant: age (41-50 years: p-value < 0.001; 51-60 years: p-value < 0.001; >60 years: p-value < 0.001), at least one comorbidity: (p-value < 0.001), the number of comorbidities (1 comorbidity: p-value < 0.001; 2 comorbidities: p-value < 0.001), and the type of comorbidity: hypertension (p-value < 0.001) and diabetes (p-value = 0.001). The proportion of severe COVID-19 status (42.1%) was estimated by considering cases with severe and critical status of COVID-19 as shown in Table 3.

**Table 3:** Bivariate analysis of severe status of COVID-19 with sociodemographic and clinical characteristics of the study participants p-values < 0.05 were considered statistically significant.

Predictor	Severe status of COVID-19		p-value
	Yes % (N)	No % (N)	
Age (in years)			
<30	10.7 (8)	89.3 (67)	
30-40	20.3 (24)	79.7 (94)	0.083
41-50	38.8 (38)	61.2 (60)	<0.001
51-60	48.5 (47)	51.5 (50)	<0.001
>60	61.9 (153)	38.1 (94)	<0.001
Gender			
Female	43.4 (129)	56.6 (168)	0.536
Male	41.0 (144)	59.0 (207)	
At least one comorbidity			
Yes	55.3 (146)	44.7 (118)	<0.001
No	33.1 (127)	66.9 (257)	
Number of comorbidities			
No	33.1 (127)	66.9 (257)	
1	51.4 (94)	48.6 (89)	<0.001
2	65.2 (45)	34.8 (24)	<0.001
>2	58.3 (7)	41.7 (5)	0.08
Types of comorbidity, HIV			
Yes	50 (7)	50 (7)	0.548
No	42.0 (266)	58.0 (368)	
Cancer			
Yes	36.4 (4)	63.6 (7)	0.697
No	42.2 (269)	57.8 (368)	
Chronic kidney disease			
Yes	66.7 (8)	33.3 (4)	0.096
No	41.7 (265)	58.3 (371)	
Hepatitis infection			
Yes	46.2 (6)	53.8 (7)	0.767
No	42.0 (267)	58.0 (368)	
Asthma			
Yes	45.8 (11)	54.2 (13)	0.702
No	41.9 (261)	58.1 (362)	
Hypertension			
Yes	61.0 (86)	39.0 (55)	<0.001
No	36.9 (187)	63.1 (320)	
Diabetes			
Yes	57.4 (58)	42.6 (43)	0.001
No	39.4 (215)	60.6 (330)	

Multivariate logistic regression analysis of factors associated with the severe status of COVID-19 on admission is detailed in Table 4.

**Table 4:** Multivariate logistic regression analysis of factors associated with severe status of COVID-19 on admission AOR: Adjusted odds ratios.

Predictors	Severe status of COVID-19		AOR (95% CI)	p-value
	Yes	No		
*Age (in years)*				
<30			Ref.	
30-40			2.20 (0.93-5.22)	0.072
41-50			5.03 (2.17-11.67)	<0.001
51-60			6.86 (2.96-15.93)	<0.001
>60			11.7 (5.35-25.67)	<0.001
*Number of comorbidities*				
No.			Ref.	
1			1.41 (0.95-2.08)	0.084
2			2.13 (1.20-3.77)	0.01
>2				
*Hypertension*				
Yes			1.03 (0.59-1.80)	0.614
No			Ref.	
*Diabetes*				
Yes			0.85 (0.46-1.56)	0.594
No			Ref.	

Table 5 shows that the mean age was 54.05 years for females and 53.05 years for males; among the patients admitted, 19.2% who died were females and 18.5% were males. The severity status of COVID-19 was at 43.4% for female patients and 41.0% for male patients. Also, the presence of comorbidity was most reported in females at 46.8% and in males at 35.6% as illustrated in Table 5.

**Table 5:** Disaggregation of gender and its predictors of COVID-19 severity

Variables	Gender		p-value
	Female % (N)	Male % (N)	
Age			
Mean ± SD	54.05 + 20.55	53.05 + 18.88	0.519
Outcome			
Died	19.2 (57)	18.5 (65)	0.827
Left hospital alive	80.8 (240)	81.5 (286)	
Status on admission			
Not severe	56.6 (168)	59.0 (207)	0.536
Severe	43.4 (129)	41.0 (144)	
Comorbidity			
Yes	46.8 (139)	35.6 (125)	0.004
No	53.2 (158)	64.4 (226)	

## Discussion

Patients of advanced age with one or more comorbidities like cardiovascular diseases, diabetes, COPD, or types of cancer are at risk of developing COVID-19 severe conditions. As the first study to show the severe status of COVID-19 and its associated factors in Rwanda, this study also revealed that old-age patients and those having two comorbidities were at risk of severe COVID-19 conditions. These findings are in line with those reported by Bajgain et al., from 27 studies worldwide, where 84.1% of fatal outcomes in countries including China, Italy, South Korea, the United States, and Iran were found in patients with one or more comorbidities [15]. A follow-up study in Egypt on 148 COVID-19 patients by Albadawy et al. reported acute respiratory distress syndrome (ARDS) at 37.8% and a three-fold risk of pneumonia in patients with comorbidities compared to those without [16]. Another study conducted in China and Ghana also reported that people of advanced age and those with comorbidities have severe COVID-19 outcomes [17,18].

Severe status was reported at 42.1%, and the CFR of 18.8% appears to be higher than that of the country's rate (1.1%) [4,11]. This study was based only on hospital admissions as most of the visiting patients had severe or moderate symptoms. Since September 2020, Rwanda introduced home-based care (HBC) to serve as an alternative for a surge of hospital cases that could not be managed at the hospital, and asymptomatic or mild cases were encouraged to self-isolate at home [4]. This has reduced the number of cases and the mortality rate across the country, adding some influencing factors such as young age and climatic factors that have been reported in the region [5,6].

Recent data have shown more case fatality among men than women with COVID-19; it also indicates that the biological factors and behavioral lifestyle of males pose a greater risk to them. This study shows that more case fatalities were in women compared to males, which might be because the mean old age and presence of comorbidity were more in females than in males. Similar studies in India have reported 2.9% case fatality in men compared to 3.3% in women [19,20]. Some risk factors were caused by exposure to infection at workplaces, where female-dominated services such as hospitality, tourism, supermarket attendants, and logistics remained open during the pandemic [20]. In Rwanda also, these services remained open during the pandemic. However, another report shows that case definitions and demographic health profiles across geographical areas may influence the COVID-19 data and gender [19].

Also, this study found that two or more comorbidity risks for severe disease were present in multivariate logistic regression analysis. However, diabetes or hypertension was not a risk factor of severity from COVID-19 after adjusting other variables. Hypertension and diabetes have been linked with COVID-19 as the most common comorbidities that appear among COVID-19 patients on admission and lead to worse outcomes. A meta-analysis of 42 studies done by Dessie and Zewotir showed that the adjusted hazard ratio of in-hospital mortality from COVID-19 among patients with hypertension and diabetes was at 1.18 and 1.17, respectively, compared to those without [21]. However, as some comorbidities tend to coexist, e.g., cardiovascular diseases, diabetes, or renal diseases, they can influence an additive risk on a bad clinical outcome of COVID-19 rather than being a lone comorbidity [22]. This study shows that 61% of patients with hypertension and 57.4% of patients with diabetes had a severe condition of COVID-19; however, the association was non-significant.

A multicenter study done by Mirza et al. on the effect of lone hypertension for severe COVID-19 status revealed that hypertension was not an independent risk factor for in-hospital death after being adjusted for other comorbidities in COVID-19 patients [23]. Although it is hard to tell, previous studies still unanswered whether the stage of hypertension or diabetes with each case is controlled or uncontrolled, or whether more than one comorbidity, stage of the disease, or its complications lead to severe condition of COVID-19 [22,24]. A prospective study done by Shalaeva et al. using multivariate logistic regression analysis reported that treated stage I and stage II hypertension did not statistically significantly increase the risk of hospitalization of COVID-19 after adjusting for confounders, but untreated stage II hypertension increased the risk of ICU admission [25]. Some comorbidities also tend to appear with advancing age, where age as a risk factor can lead to the added risk of severe outcomes. In Rwanda, for example, NCD mortality was at 63% among the age groups above 40 years in 2013 [26].

In contrast to a study conducted in Nigeria by Osibogun et al. [14], this study shows that other comorbidities including HIV, cancer, asthma, CKD, and hepatitis did not affect the severe status of COVID-19. The compromised immune system has also been related to severe outcomes of COVID-19 in previous literature, where diseases like HIV, hepatitis, and CKD pathogenesis interfere with normal body immune regulation and lead to the worsening of COVID-19 conditions [9]. A study done by Iradukunda et al. showed that COVID-19 did not predispose HIV individuals at high risk to anti-retro therapy (ART) with their viral load suppressed [27], and a study on patients with controlled asthma in the United Kingdom and patients on scheduled cancer regimen displayed no severe COVID-19 outcomes [28,29]. In Rwanda, specialized care for people with underlying conditions continued to be available irrespective of their COVID-19 status during the pandemic, and outpatient visits continued at designated centers while screening for COVID-19 symptoms. Also, hemodialysis services for renal patients, oncology services, and ART services were offered by the multidisciplinary team at the treatment center. Special cases were transferred for special service to another health entity by healthcare personnel and then returned to the treatment center while respecting COVID-19 infection prevention and control (IPC) measures. 

Although few studies are available based on comorbidity treatment modality in COVID-19 patients, one study in the United States among renal transplant patients taking immune-suppressant medications found that these patients did not have COVID-19 severe symptoms [13]. In Rwanda, HIV/AIDS has been kept at a low prevalence across the country (3%) [27]; this may be because of early taking of ART regardless of the CD4 cell count, which reduced the prevalence of AIDS in Rwanda. Also, a recent study in Rwanda has shown high knowledge and practice score regarding COVID-19 preventive measures among people living with HIV [27]. During the pandemic, case follow-up was done aggressively by the Rwanda Ministry of Health through the use of technology and home visits to people with advanced age and comorbidities; this has played a big role in reducing the severity of COVID-19 symptoms [11,30].

The strength of this study is that it was based on hospital medical records based on a big number of patients. However, there are also some limitations concerning the study; one of the limitations is that comorbidities were based on self-reporting, which could underestimate the true burden of comorbidity since some are not diagnosed yet. Another limitation is that the results focused on hospitalized patients only, which may exclude other patients who were not in the hospital from the study. Also, some data may be missing, and confounding factors such as nutrition, stage of comorbidity, or change in lifestyles are not documented.

## Conclusions

According to the findings of this study, people of old age and those with two or more comorbidities are likely to develop a risk of severe COVID-19 status compared to those who do not. Therefore, vaccination efforts should be prioritized in this vulnerable population.
